# Colocalization of somatostatin receptors and epidermal growth factor receptors in breast cancer cells

**DOI:** 10.1186/1475-2867-6-5

**Published:** 2006-03-06

**Authors:** Heather L Watt, Ujendra Kumar

**Affiliations:** 1 Fraser Laboratories For Diabetes Research, Department of Medicine, Royal Victoria Hospital, McGill University, Montreal, Quebec, H3A 1A1, Canada; 2 Faculty of Pharmaceutical Sciences, Division of Pharmacology and Toxicology, The University of British Columbia, Vancouver, BC, Canada

## Abstract

**Background:**

Somatostatin receptor (SSTR) expression is positively correlated with tumor size and inversely correlated with epidermal growth factor receptor (ErbB) levels and tumor differentiation. In the present study, we compared SSTR1-5 and ErbB1-4 mRNA and protein expression in two breast cancer cell lines: MCF-7 (ER+) and MDA-MB-231 (ERα-).

**Results:**

All five SSTRs and four ErbBs were variably expressed as both cell surface and cytoplasmic proteins. In both cell lines, SSTR4 and SSTR1 were highly expressed, followed by SSTR2 and SSTR5 with SSTR3 being the least expressed subtype, at the protein level. ErbBs were variably expressed with ErbB1 as the predominant subtype in both cell lines. ErbB1 is followed by ErbB3, ErbB2 and ErbB4 in MCF-7 at both the protein and mRNA levels. In MDA-MB-231 cells, ErbB1 is followed by ErbB2, ErbB4 and ErbB3. Our results indicate significant correlations at the level of mRNA and protein expression in a cell and receptor-specific manner. Using indirect immunofluorescence, we found that, in MCF-7 cells, SSTR5 was the most prominent subtype coexpressed with ErbBs followed by SSTR3, SSTR4, SSTR1 and SSTR2, respectively. In MDA-MB-231 cells, SSTR1 colocalized strongly with ErbBs followed by SSTR5, SSTR4, SSTR3 and SSTR2. ErbBs displayed higher levels of colocalization amongst themselves in MCF-7 cells than in MDA-MB-231 cells.

**Conclusion:**

These findings may explain the poor response to endocrine therapy in ER-cancer. Differential distribution of SSTR subtypes with ErbBs in breast cancer cells in a receptor-specific manner may be considered as a novel diagnosis for breast tumors.

## Background

Somatostatin (SST) is an endogenously produced peptide in neuroendocrine and immune cells. It exists as two biologically active forms, SST-14 and SST-28, which are produced by tissue-specific proteolytic processing of a common precursor [1]. SST is a potent inhibitor of hormone and growth factor secretion as well as a modulator of cell proliferation [2,3]. These actions are mediated by a family of G protein-coupled receptors (GPCR) with five known subtypes (SSTR1-5). SST exerts antiproliferative effects on normal dividing cells, such as intestinal mucosal cells, activated lymphocytes and inflammatory cells as well as on solid tumors and cultured cells derived from both endocrine and epithelial tumors. These effects include cytostatic (growth arrest) and cytotoxic (apoptotic) actions and are mediated (i) directly by SSTRs present on tumor cells, and (ii) indirectly via SSTRs present on non-tumor cell targets. SST inhibits the secretion of hormones and growth factors that promote tumor growth, inhibits growth factor-induced DNA synthesis, inhibits angiogenesis, promotes vasoconstriction and modulates immune cell function [1]. Moreover, immunoreactive SST has been identified, by immunohistochemistry, in 30% of breast cancer samples and in several breast cancer cell lines [4,5]. Whether SST is synthesized and secreted from these cells and acts as a paracrine/autocrine growth inhibitor remains to be established.

All five SSTRs have been implicated in antiproliferative signaling in a subtype selective manner. When studied as individual isotypes, four of the receptors (SSTR1, 2, 4, 5) induce cell cycle arrest whereas SSTR3 uniquely triggers apoptosis [3,6]. Previous studies have demonstrated the presence of SSTRs in a large variety of tumors and cancer cell lines [7-9]. In addition, 15–66% of primary human breast tumors are SSTR-positive by binding analysis [10-14]. Consistent with previous studies, we have recently shown that SSTRs are expressed in breast cancers in variable amounts and are correlated with various histological markers in a receptor-specific manner [15]. We have also shown the effects of estradiol and tamoxifen on SSTR1 and SSTR2 expression in breast cancer cells [16].

Epidermal growth factor receptors, members of the type I receptor tyrosine kinase (RTK) family commonly known as ErbBs, are also variably distributed in breast tumors and breast cancer cell lines as are SSTRs [17,18]. ErbBs can be detected in all tumors with variable degrees of expression. There are currently four known ErbB receptors with ErbB1 (also known as EGFR) and ErbB2 (also known as Neu or HER2) being the most likely to be overexpressed in cancers, and, therefore, the most studied [19-22]. ErbB3 and ErbB4 (also known as HER3 and HER4, respectively) have been investigated the least. ErbBs exist as monomers and, upon ligand activation or when overexpressed, form homo- and heterodimers [23,24].

Previous studies showed that ErbB1 is expressed in 40–50% of breast cancer cases and is inversely related with estrogen receptor (ER) levels and survival [25-27]. This is associated with more aggressive proliferation and unresponsiveness to hormone treatment [12,14,27]. Similarly, ErbB2 is present in 10–40% of breast cancer cases and is associated with poor survival [19,21,25,26]. ErbB3 is also expressed in breast cancer [28,29]. Associations with ErbB1 and ER have been shown in some studies but not in others [20]. This discrepancy may be due to the techniques employed, antibodies used, sample size or tumor type. In contrast with ErbB1-3, ErbB4 is generally reported to be associated with favorable prognostic factors [20,21,25,30,31].

While ErbBs are involved in tumor growth and cell proliferation and are often associated with poor response to endocrine therapy and reduced survival, SSTRs play a major role in the control of tumor growth and tumor cell proliferation [32-34]. SSTR expression is positively correlated with tumor size and inversely correlated with ErbB levels and tumor differentiation [12,14]. Several recent reports have shown GPCRs to directly interact with RTKs via scaffolding proteins when both receptors are present together in the large signaling complexes [35-37]. Alternatively, GPCRs can indirectly transactivate RTKs via G proteins which ultimately lead to increased intracellular calcium levels and activation of PKC [38]. Indirect RTK transactivation has also been reported to occur via membrane-bound metalloproteinases (MMPs) or metalloproteinase-disintegrin proteins (ADAMs) which process ErbB transmembrane ligands [35,39,40]. In general, RTK transactivation by GPCRs results in altered mitogen activated protein kinase (MAPK) signaling and, subsequently, in altered cell growth and proliferation [39,41,42]. It is not known if SSTRs (GPCR) and ErbBs (RTK) are coexpressed within the same cells. Hence, before defining the mechanisms for functional interactions between ErbBs and SSTRs, it is essential to determine if this occurs. We have therefore determined, in the current study, SSTR1-5 and ErbB1-4 expression at the protein and mRNA levels. In addition, since ER has been shown to be associated with ErbB levels, we investigated their colocalization in ER-positive (ER+) and negative (ER-) breast cancer cells. Our data showed that SSTRs and ErbBs are well expressed in both cell lines and, significantly, exhibited variable colocalization.

## Results

### Expression of SSTRs mRNA and protein in MCF-7 and MDA-MB-231 cells

**Figure 1:**
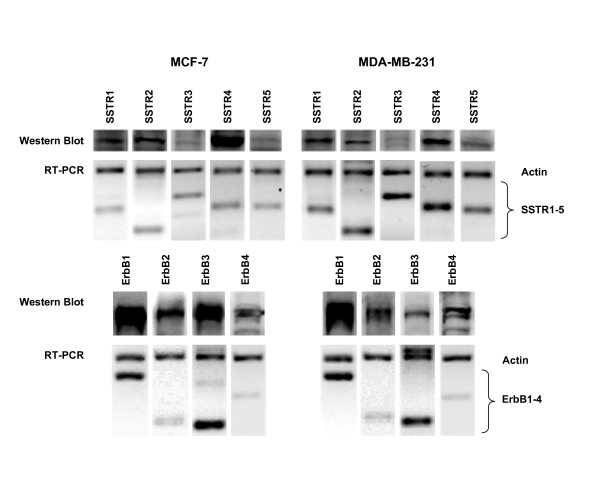
Semi-quantitative analysis of SSTR1-5 and ErbB1-4 mRNA and protein expression in MCF-7 and MDA-MB-231 breast cancer cells. A. Upper panel shows western blot analysis of SSTR1-5 in MCF-7 (left) and MDA-MB-231 (right) cells. Membrane protein (25 μg) was fractionated by SDS-PAGE and probed with affinity-purified SSTR antibodies. Major protein bands of 53 (SSTR1), 57 (SSTR2), 60 (SSTR3), 44 (SSTR4) and 58 kDa (SSTR5) were obtained. Lower panel shows RT-PCR anlaysis of SSTR1-5 mRNA expression in both cell lines. 5 μg of DNA-free RNA was reverse transcribed and coamplified with primers specific for SSTR1-5 and β-actin. 8 μL of PCR products were fractionated on agarose gels stained with ethidium bromide, visualized under UV lighting and photographed. B. Western blot (upper panel) and RT-PCR (lower panel) analysis of ErbB1-4 expression in MCF-7 (left) and MDA-MB-231 (right) breast tumor cells. Major protein bands of 170 (ErbB1), 185 (ErbB2), 200 (ErbB3) and 175 kDa (ErbB4) were obtained. Experimental conditions were the same as described for panel A except for the specific antibodies and primers.

Using semi-quantitative RT-PCR, we determined SSTR1-5 mRNA expression in MCF-7 (ER+) and MDA-MB-231 (ERα-) human breast cancer cells (Fig. 1A). We found significant differences in overall receptor expression levels between ER+ and ERα – cells. Although SSTR mRNA levels were greater in MDA-MB-231 than in MCF-7 cells, both cells lines showed similar patterns of expression. SSTR3 was highly expressed, followed by SSTR4, SSTR2 and SSTR5 while SSTR1 was the least expressed subtype, at the level of the mRNA.

**Figure 2:**
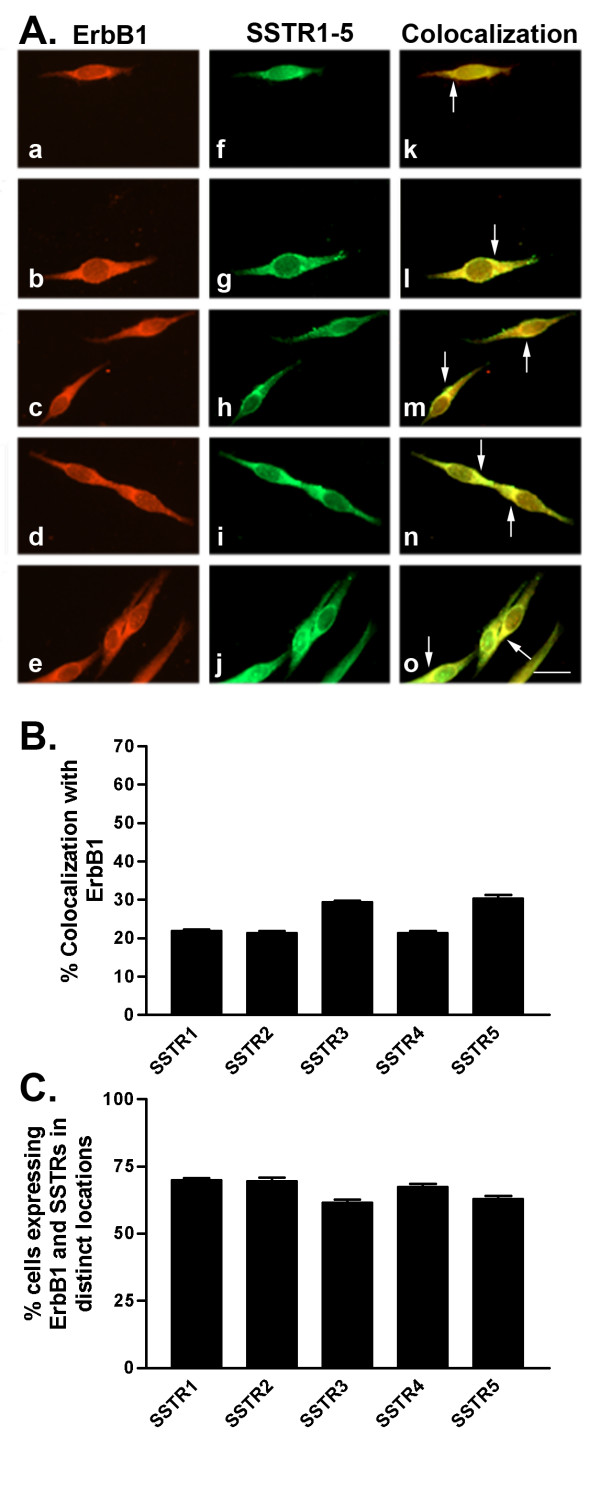
A. Representative photomicrographs illustrating double immunofluorescence localization of ErbB1 and SSTR1-5 in MCF-7 cells. Localization of ErbB1 (red staining) was visualized using monoclonal antibodies with Cy3-conjugated goat anti-mouse IgG (a-e). The same cells were incubated with polyclonal SSTR1-5 antibodies and visualized (green staining) using FITC-conjugated goat anti-rabbit IgG (f-j). Colocalization of ErbB1 and SSTR1-5 was determined by merging individual red and green images to give orange-labelled cells (k-o). All receptors are expressed as membrane and cytoplasmic protein. Arrows indicate colocalization at the cell surface. Scale bar = 25 μm. B. Quantitative analysis of MCF-7 cells showing colocalization of ErbB1 with SSTR1-5. Cells expressing two receptors together were counted from at least 8 randomly selected vertical and horizontal fields from each coverslip. Data are from three different experiments performed in duplicate and are presented as mean ± SEM for each receptor combination. C. Quantitative analysis of cells showing ErbB1 and SSTR1-5 in distinct locations within the same cell. Data were analyzed as described in B.

**Figure 3:**
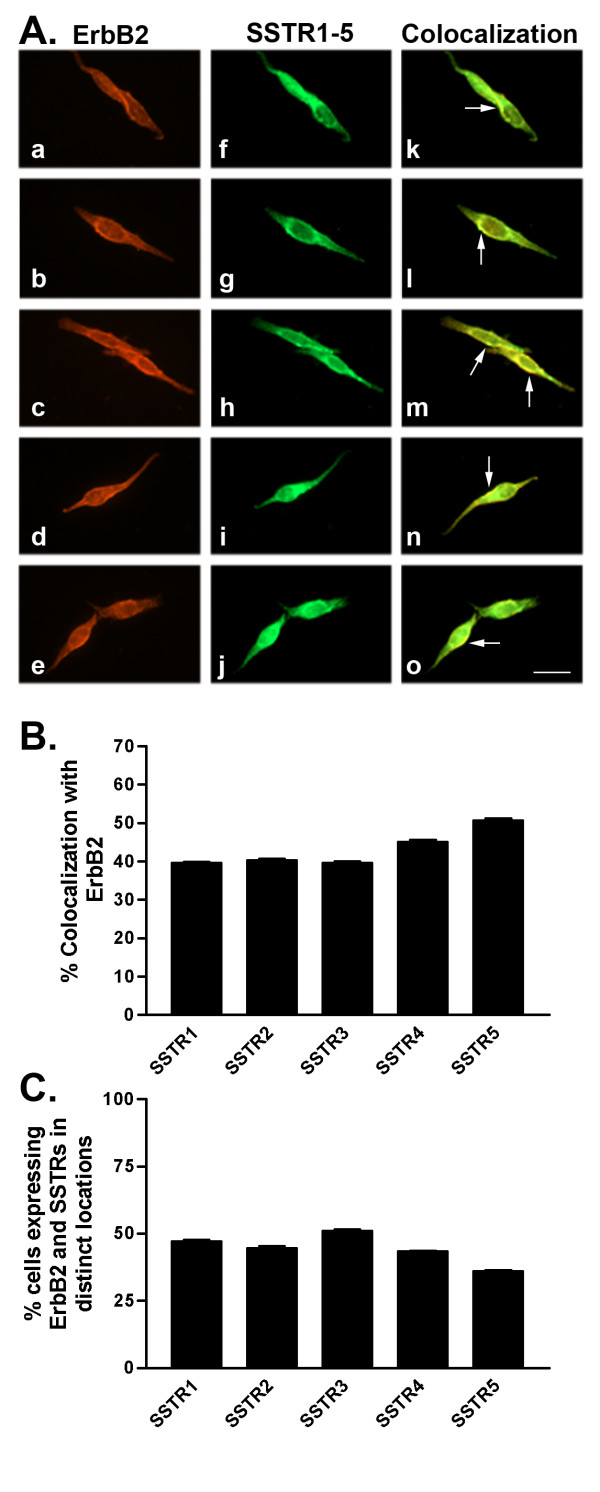
A. Representative photomicrographs illustrating double immunofluorescence localization of ErbB2 (red staining) and SSTR1-5 (green staining) in MCF-7 cells (for details see legend to Figure 2). Scale bar = 25 μm. B. Quantitative analysis of MCF-7 cells showing colocalization of ErbB2 with SSTR1-5 (for details see legend to Figure 2). C. Quantitative analysis of cells showing ErbB2 and SSTR1-5 in distinct locations within the same cell. Data were analyzed as described in Figure 2.

**Figure 4:**
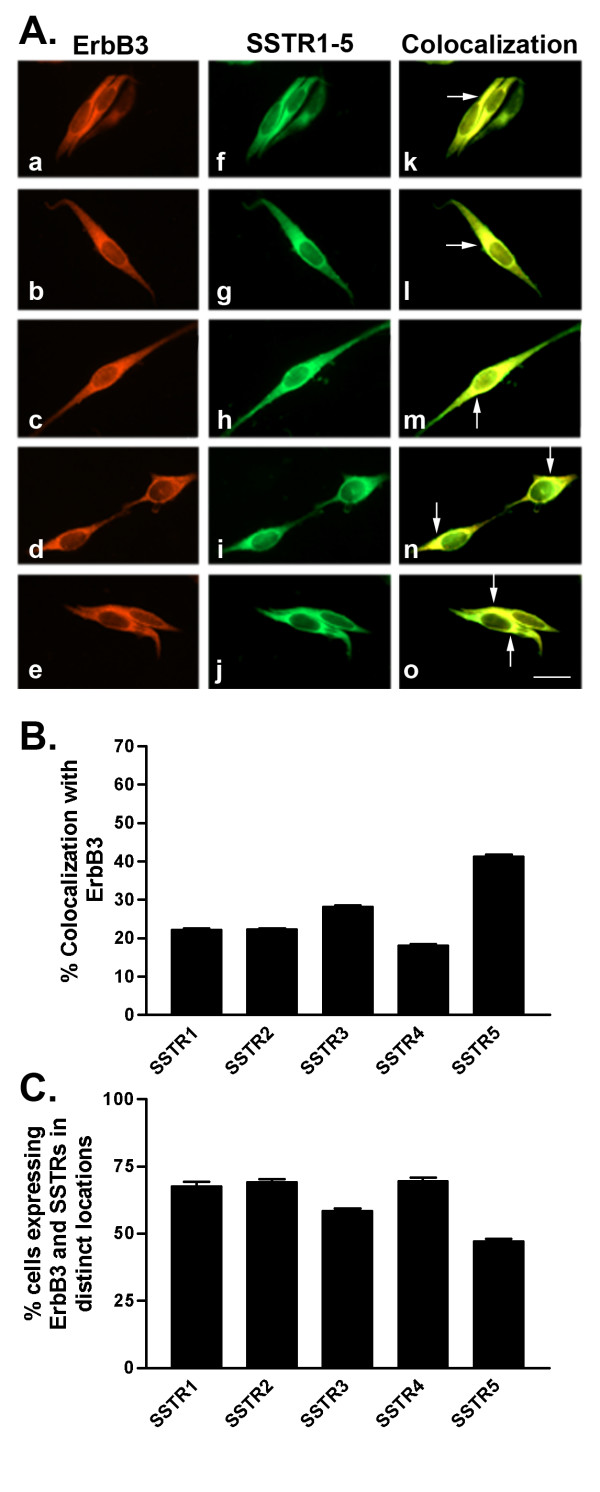
A. Representative photomicrographs illustrating double immunofluorescence localization of ErbB3 (red staining) and SSTR1-5 (green staining) in MCF-7 cells (for details see legend to Figure 2). Scale bar = 25 μm. B. Quantitative analysis of MCF-7 cells showing colocalization of ErbB3 with SSTR1-5 (for details see legend to Figure 2). C. Quantitative analysis of cells showing ErbB3 and SSTR1-5 in distinct locations within the same cell. Data were analyzed as described in Figure 2.

**Figure 5:**
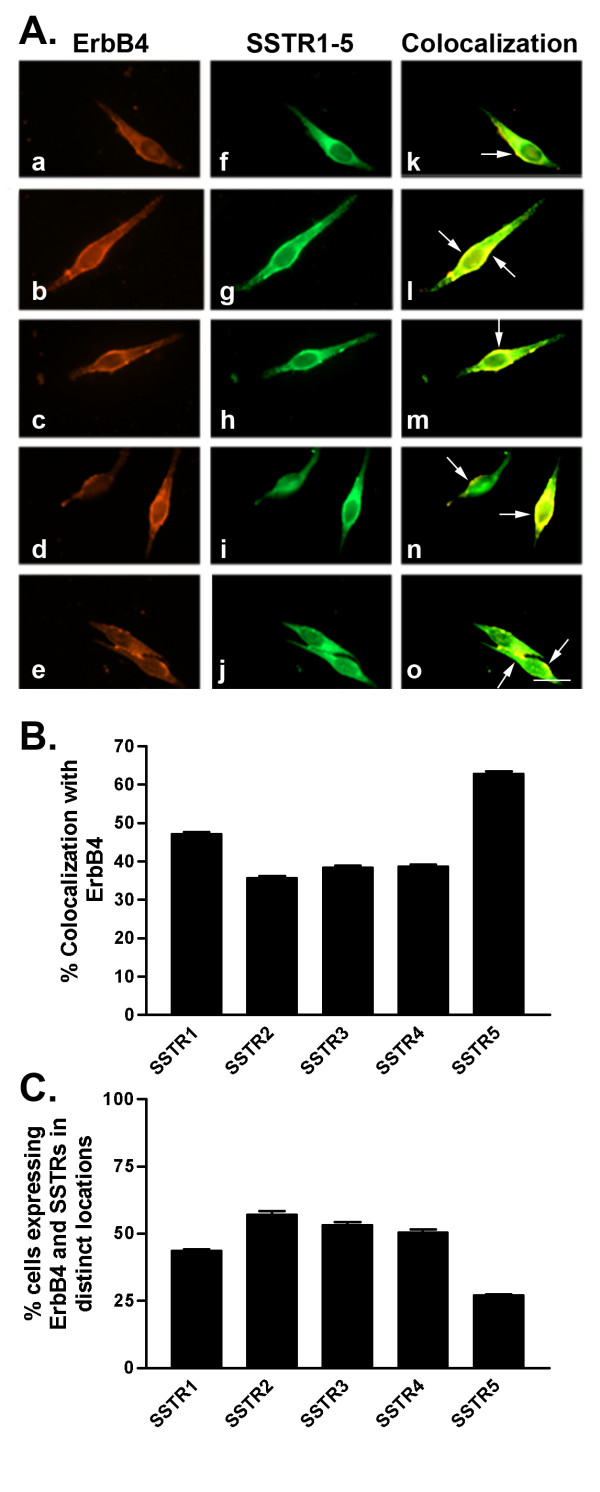
A. Representative photomicrographs illustrating double immunofluorescence localization of ErbB4 (red staining) and SSTR1-5 (green staining) in MCF-7 cells (for details see legend to Figure 2). Scale bar = 25 μm. B. Quantitative analysis of MCF-7 cells showing colocalization of ErbB4 with SSTR1-5 (for details see legend to Figure 2). C. Quantitative analysis of cells showing ErbB4 and SSTR1-5 in distinct locations within the same cell. Data were analyzed as described in Figure 2.

**Figure 6:**
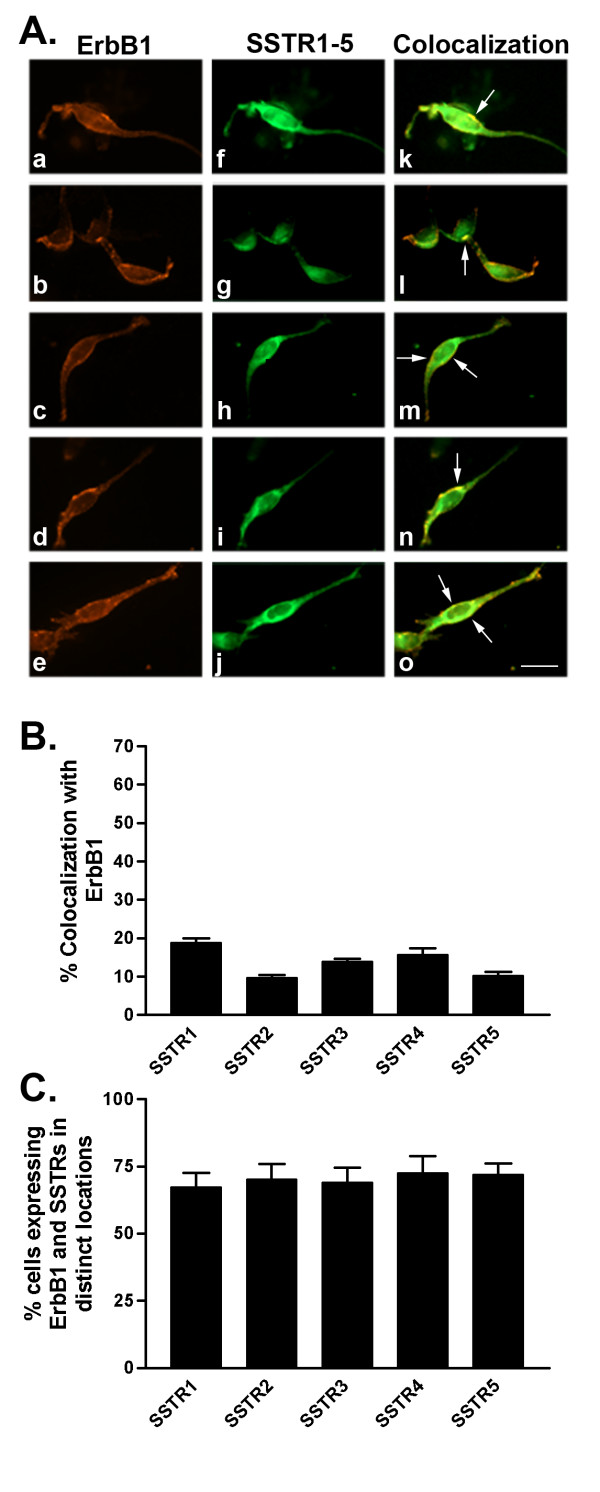
A. Representative photomicrographs illustrating double immunofluorescence localization of ErbB1 (red staining) and SSTR1-5 (green staining) in MDA-MB-231 cells (for details see legend to Figure 2). Scale bar = 25 μm. B. Quantitative analysis of MDA-MB-231 cells showing colocalization of ErbB1 with SSTR1-5 (for details see legend to Figure 2). C. Quantitative analysis of cells showing ErbB1 and SSTR1-5 in distinct locations within the same cell. Data were analyzed as described in Figure 2.

**Figure 7:**
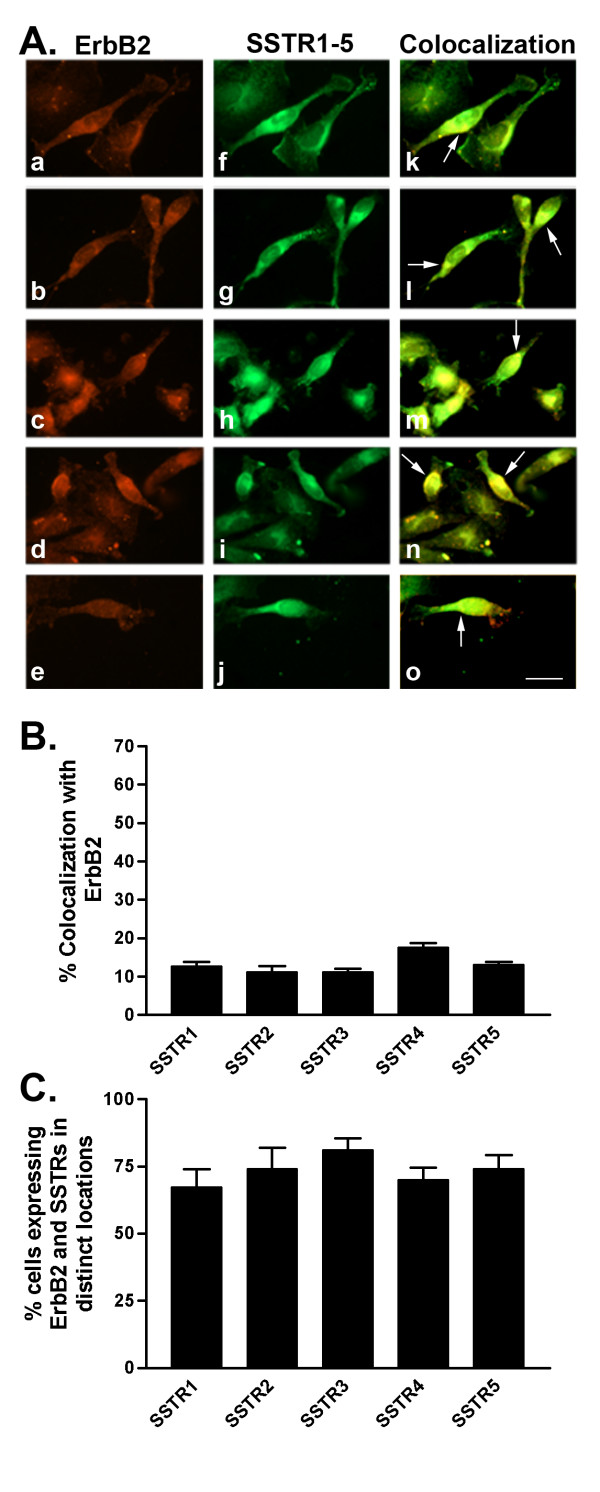
A. Representative photomicrographs illustrating double immunofluorescence localization of ErbB2 (red staining) and SSTR1-5 (green staining) in MDA-MB-231 cells (for details see legend to Figure 2). Scale bar = 25 μm. B. Quantitative analysis of MDA-MB-231 cells showing colocalization of ErbB2 with SSTR1-5 (for details see legend to Figure 2). C. Quantitative analysis of cells showing ErbB2 and SSTR1-5 in distinct locations within the same cell. Data were analyzed as described in Figure 2.

**Figure 8:**
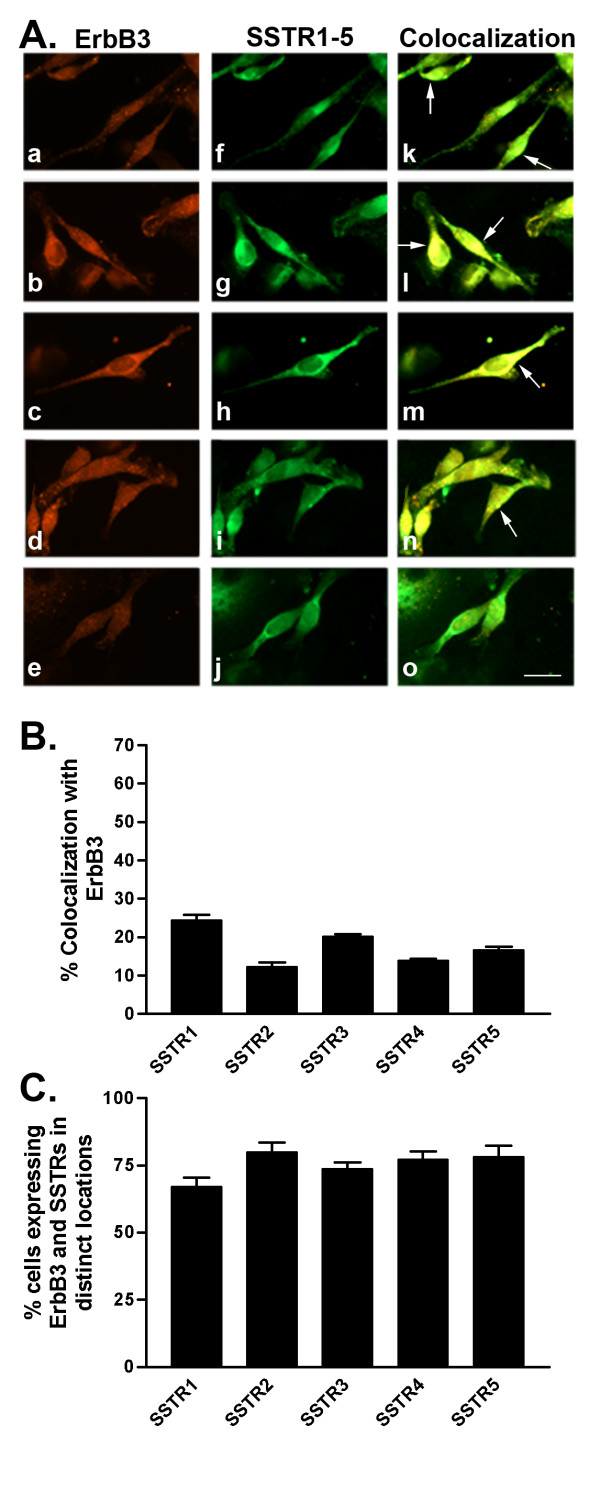
A. Representative photomicrographs illustrating double immunofluorescence localization of ErbB3 (red staining) and SSTR1-5 (green staining) in MDA-MB-231 cells (for details see legend to Figure 2). Scale bar = 25 μm. B. Quantitative analysis of MDA-MB-231 cells showing colocalization of ErbB3 with SSTR1-5 (for details see legend to Figure 2). C. Quantitative analysis of cells showing ErbB3 and SSTR1-5 in distinct locations within the same cell. Data were analyzed as described in Figure 2.

**Figure 9:**
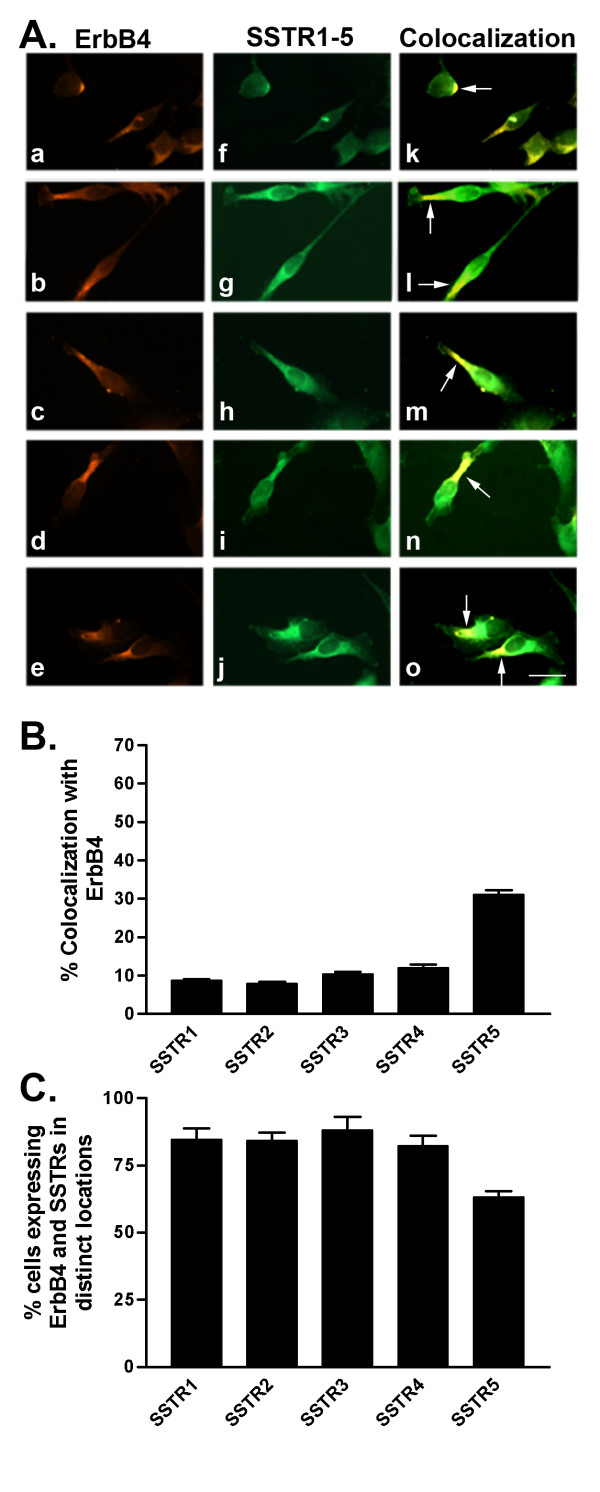
A. Representative photomicrographs illustrating double immunofluorescence localization of ErbB4 (red staining) and SSTR1-5 (green staining) in MDA-MB-231 cells (for details see legend to Figure 2). Scale bar = 25 μm. B. Quantitative analysis of MDA-MB-231 cells showing colocalization of ErbB4 with SSTR1-5 (for details see legend to Figure 2). C. Quantitative analysis of cells showing ErbB4 and SSTR1-5 in distinct locations within the same cell. Data were analyzed as described in Figure 2.

We further determined SSTR1-5 protein expression using western blot and indirect immunofluorescence analyses. Consistent with mRNA results and as detected by western blot, all SSTR subtypes were expressed at their representative molecular sizes at the protein level (53, 57, 60, 44 and 58 kDa for SSTR1-5, respectively) (Table 1 and Fig. 1A). Indirect immunofluorescence analysis of SSTR subtypes revealed a significant but variable cellular expression of multiple SSTRs with all five receptor subtypes expressed as both membrane and cytoplasmic proteins (Figs. 2, 3, 4, 5, 6, 7, 8, 9). Notably, SSTR1 and 4 were more highly expressed in MCF-7 cells than in MDA-MB-231 cells while SSTR3 was poorly expressed in both cell lines.

**Table 1:** Semiquantitative analysis of relative protein expression levels of SSTR1-5 in MCF-7 and MDA-MB-231 cells as determined by western blot analysis.

	**MCF-7**	**MDA-MB-231**
**SSTR1**	++	+++
**SSTR2**	+++	++
**SSTR3**	+	+
**SSTR4**	++++	++++
**SSTR5**	+	+

++++ strong +++ moderate ++ mild + weak

### Expression of ErbBs mRNA and protein in MCF-7 and MDA-MB-231 cells

All ErbB subtypes are well expressed at the mRNA level in a significant proportion of breast tumor tissues; however, expression in breast cancer cells is variable [22,43,44]. MCF-7 cells expressed all four ErbBs at the level of the mRNA with ErbB1 and ErbB3 being the dominant subtypes (Fig. 1B). MDA-MB-231 cells expressed all four ErbBs in a comparable manner, also displaying higher expression at the mRNA level for ErbB1 and ErbB3. Interestingly, in MCF-7 cells, ErbB3 mRNA expression was the strongest while, in MDA-MB-231 cells, ErbB1 mRNA was the most abundant. These results are in agreement with a report by Bièche et al. [43] where MCF-7 cells displayed lower ErbB1 mRNA levels, higher ErbB2 and ErbB3 levels and equivalent ErbB4 mRNA expression in comparison to MDA-MB-231 cells.

**Figure 10:**
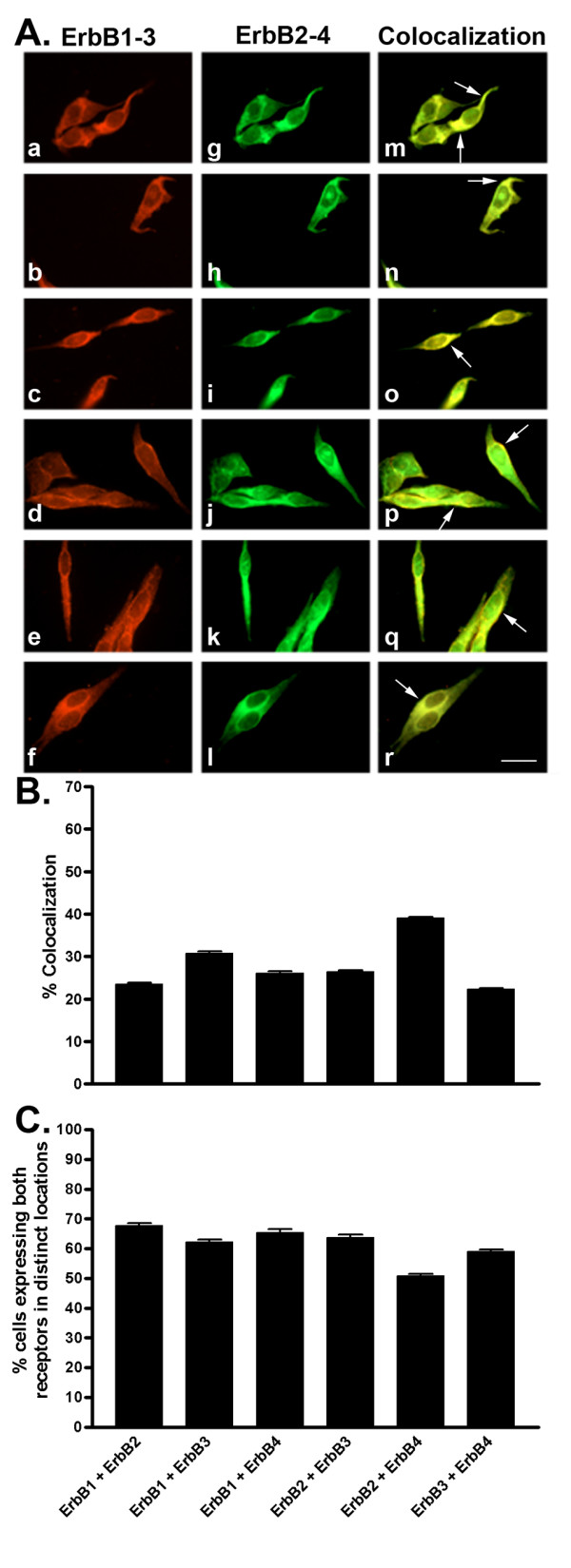
A. Representative photomicrographs illustrating double immunofluorescence localization of ErbB1-3 and ErbB2-4 in MCF-7 cells. Localization of ErbB1-3 (red staining) was visualized using monoclonal antibodies with Cy3-conjugated goat anti-mouse IgG (a-f). The same cells were incubated with polyclonal ErbB2-4 antibodies and visualized (green staining) using FITC-conjugated goat anti-rabbit (g-l). Colocalization of ErbB1-3 and ErbB2-4 was determined by merging individual red and green images to give orange-labelled cells (m-r). All receptors are expressed as membrane and cytoplasmic protein. Arrows indicate colocalization at the cell surface. Scale bar = 25 μm. B. Quantitative analysis of MCF-7 cells showing colocalization of ErbB1-3 with ErbB2-4 (for details see legend to Figure 2). C. Quantitative analysis of cells showing ErbB1-3 and ErbB2-4 in distinct locations within the same cell. Data were analyzed as described in Figure 2.

**Figure 11:**
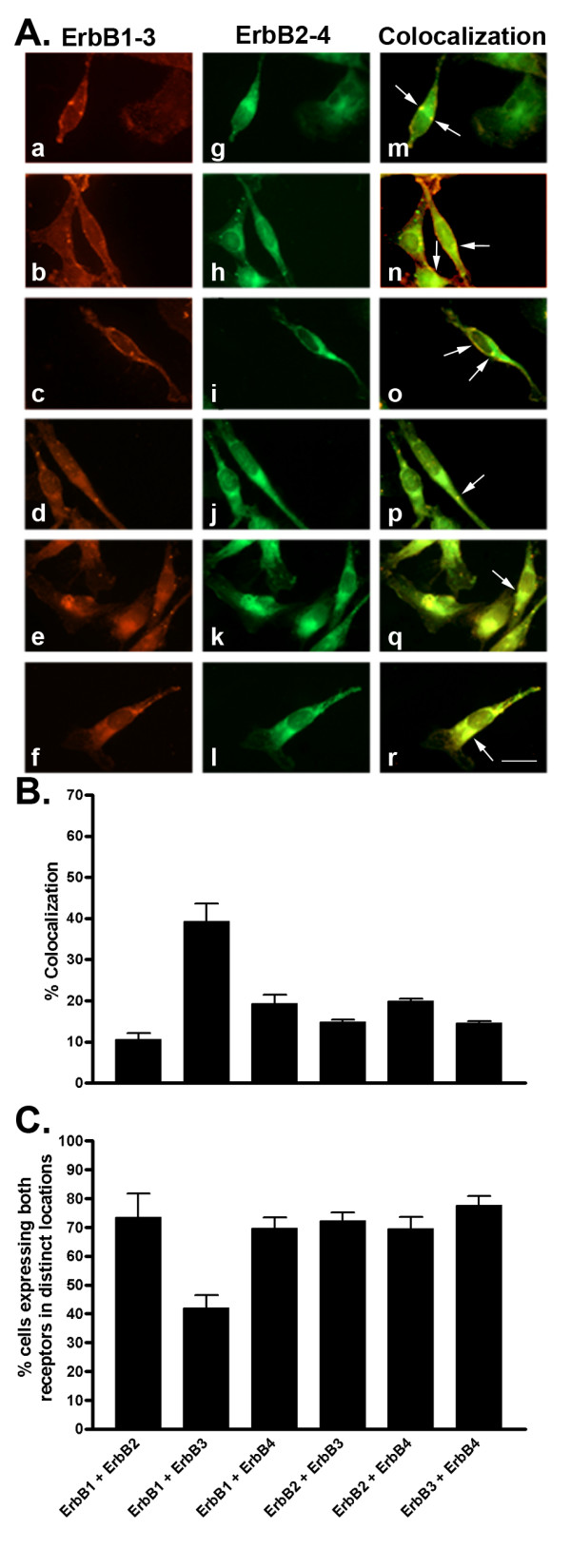
A. Representative photomicrographs illustrating double immunofluorescence localization of ErbB1-3 and ErbB2-4 in MDA-MB-231 cells (for details see legend to Figure 10). Scale bar = 25 μm. B. Quantitative analysis of MDA-MB-231 cells showing colocalization of ErbB1-3 with ErbB2-4 (for details see legend to Figure 2). C. Quantitative analysis of cells showing ErbB1-3 and ErbB2-4 in distinct locations within the same cell. Data were analyzed as described in Figure 2.

Using western blot analysis, ErbB subtypes in MCF-7 and MDA-MB-231 cells displayed variable expression at the protein level whereby all ErbBs were expressed at their representative molecular sizes (170, 185, 200 and 175 kDa for ErbB1-4, respectively). ErbB1 and ErbB3 were the predominant subtypes followed by ErbB2 and ErbB4 in MCF-7 cells as determined by western blot analysis (Table 2 and Fig. 1B). In contrast, in MDA-MB-231 cells, ErbB1 was predominantly expressed followed by ErbB2, ErbB4 and ErbB3. Consistent with previous reports, ErbB3 protein expression was strongest in ER+ cells while ErbB1 was more abundant in ER-cells [22]. However, our results contradict another report with regards to relative ErbB3 expression levels [28]. Protein expression was further confirmed by immunocytochemistry revealing that all ErbB subtypes were well expressed as membrane and cytoplasmic proteins in MCF-7 and MDA-MB-231 cells (Figs. 2, 3, 4, 5, 6, 7, 8, 9, 10, 11).

**Table 2:** Semiquantitative analysis of relative protein expression levels of ErbB1-4 in MCF-7 and MDA-MB-231 cells as determined by western blot analysis.

	**MCF-7**	**MDA-MB-231**
**ErbB1**	++++	++++
**ErbB2**	++	+++
**ErbB3**	+++	+
**ErbB4**	++	++

++++ strong +++ moderate ++ mild + weak

### Colocalization of SSTRs and ErbBs in MCF-7 cells

Colocalization between SSTRs and ErbBs revealed significant variations in a receptor and cell-specific manner. Four different cell populations were detected in MCF-7 cells: one expressing SSTRs alone (≤ 7%), a second population expressing only ErbBs (≤ 15%), a third population expressing both receptors in distinct locations within the same cell (27–70%) and a fourth population of cells displaying colocalization (18–62%).

In MCF-7 cells, SSTR1 colocalized with ErbB1 (22% of cells) at the cell membrane and intracellularly (Table 3 and Fig. 2). SSTR2 and SSTR4 exhibited similar patterns of colocalization with ErbB1 with only 21% of cells coexpressing both receptors. SSTR3 and SSTR5 colocalized with ErbB1 in a greater proportion (30%) of cells. All SSTR subtypes colocalized with ErbB2 in a comparable manner at the cell surface as well as intracellularly (Table 3 and Fig. 3). ErbB2 and SSTRs colocalized in 40–51% of cells with SSTR5 displaying the strongest colocalization with ErbB2. ErbB3 was coexpressed with SSTR1-5 in a comparable manner to ErbB1 (Table 3 and Fig. 4). SSTR1 and SSTR2 colocalized with ErbB3 in 22% of cells whereas SSTR3 and SSTR4 were coexpressed in 28 and 18% of ErbB3-positive cells, respectively. In contrast, SSTR5 colocalized with ErbB3 in about 41% of cells. In MCF-7 cells, ErbB4 colocalized with all SSTR subtypes (Table 3 and Fig. 5). ErbB4 was coexpressed with SSTR2, SSTR3, and SSTR4 in a comparable manner (36–39% of cells). On the other hand, SSTR1 and SSTR5 colocalized with ErbB4 in 47 and 63% of cells, respectively. Further colocalization studies revealed that SSTR5 was the most prominent SSTR subtype to colocalize with ErbB1-4 in MCF-7 cells (Table 3 and Figs. 2, 3, 4, 5).

**Table 3:** Colocalization of SSTR1-5 with ErbB1-4 in MCF-7 and MDA-MB-231 cells.

	**MCF-7**				**MDA-MB-231**			
	**ErbB1**	**ErbB2**	**ErbB3**	**ErbB4**	**ErbB1**	**ErbB2**	**ErbB3**	**ErbB4**
**SSTR1**	+	++	+	+++	++	+	+++	+
**SSTR2**	+	++	+	++	+	+	+	+
**SSTR3**	+++	++	++	++	++	+	++	+
**SSTR4**	+	+++	+	++	++	++	+	+
**SSTR5**	+++	+++	+++	++++	+	+	++	+++

### Colocalization of SSTRs and ErbBs in MDA-MB-231 cells

In comparison with MCF-7 (ER+) cells, MDA-MB-231 (ERα-) cells exhibited significantly variable colocalization of SSTR1-5 with ErbB1-4. Furthermore, a lower percentage of cells coexpressed both SSTRs and ErbBs in MDA-MB-231 than in MCF-7 cells. Interestingly, using immunocytochemistry, 100% of MDA-MB-231 cells expressed ErbB1-3. Subsequently, there were no cells that only expressed SSTRs when the cells were double-labeled for SSTRs and ErbB1-3. In contrast, up to 3% of cells showed staining for SSTRs alone while up to 20% of cells only expressed ErbB4 in cells double-labeled for SSTR1-5 and ErbB4. Furthermore, there was a small cell population (≤ 1%) lacking both receptors.

As illustrated in Table 3 and Figure 6, 19% of MDA-MB-231 cells displayed strong colocalization between SSTR1 and ErbB1. On the other hand, SSTR2-5 colocalization with ErbB1 occurred in only 10–16% of cells (Table 3 and Fig. 6). In MDA-MB-231 cells, ErbB2 weakly colocalized with all SSTR subtypes at the cell surface in only 11–18% of cells (Table 3 and Fig. 7). SSTR1 was coexpressed with ErbB3 in 24% of cells (Table 3 and Fig. 8). SSTR2, SSTR3 and SSTR4 colocalized with ErbB3 at the cell surface and intracellularly in approximately 12, 20 and 14%, respectively, of the cell population (Table 3 and Fig. 8). Meanwhile, SSTR5 displayed colocalization (17% of cells) with ErbB3 mainly at the cell surface. In MDA-MB-231 cells, SSTR1-4 colocalized with ErbB4 at the cell surface in 8–12% of cells (Table 3 and Fig. 9). In contrast, SSTR5 and ErbB4 colocalization was seen in 31% of cells. Notably, colocalization of SSTRs with ErbB4 occurred mainly in the "apical" endings of the cells.

### Colocalization of ErbBs in MCF-7 cells and MDA-MB-231 cells

To better understand whether there is any preferential and selective colocalization between ErbB subtypes in ER+ and ER-cells, we determined the colocalization of ErbBs in MCF-7 and MDA-MB-231 cells. As shown in MCF-7 cells (Fig. 10), ErbB2, ErbB3 and ErbB4 colocalized with ErbB1 in 23%, 31% and 26% of cells, respectively. Furthermore, ErbB3 and ErbB4 were coexpressed with ErbB2 in 26% and 39%, respectively, while ErbB3 and ErbB4 colocalized in 22% of MCF-7 cells. In contrast, MDA-MB-231 cells demonstrated lesser degrees of colocalization than MCF-7 cells with the exception of ErbB1 and ErbB3 (Fig. 11). ErbB1 colocalized with ErbB2, ErbB3 and ErbB4 in 11%, 39% and 19% of cells, respectively. Meanwhile, ErbB3 and ErbB4 colocalized with ErbB2 in 15% and 20% of cells, respectively, and ErbB3 and ErbB4 were coexpressed in 14% of the cell population.

## Discussion

The present study represents the first comprehensive description showing SSTR1-5 and ErbB1-4 colocalization in ER+ and ER-breast cancer cells. All five SSTRs were detected in MCF-7 and MDA-MB-231 with a rich expression of subtypes 1 and 4, moderate expression of SSTR2 and relatively weak expression of subtypes 3 and 5. Our data also demonstrate a potential correlation between SSTR and ErbB expression and estrogen dependency. We found higher levels of expression of ErbB1 and lower levels of SSTR1, SSTR4 and ErbB3 in ERα – (MDA-MB-231) cells when compared to ER+ (MCF-7) breast cancer cells. In addition, we showed that there was more colocalization of SSTRs with ErbBs in MCF-7 cells than in MDA-MB-231 cells. We also detected preferential colocalization among ErbBs in both MCF-7 and MDA-MB-231 cells.

Overall expression levels of SSTR subtypes in cultured breast cancer cell lines were comparatively less than in solid tumors. Significantly, SSTR3, which is well expressed in breast tumor tissues, was relatively poorly expressed in these cell lines [15]. These results indicate that the various breast cancer cell lines, although useful for studying SSTR biology, do not necessarily reflect endogenous tumor SSTR expression or function. Possible explanations for the difference are the probable induction of SSTR expression in solid tumors by circulating hormones, or, locally, by growth factors, cytokines, and other mediators produced from peritumoral structures such as the stroma, blood vessels and immune cells [45]. Increasing evidence points to the occurrence of multiple SSTR subtypes in many different types of tumor cells as well as normal cells [46,47]. All five SSTR isoforms bind the natural ligands SST-14 and SST-28 with nanomolar affinity and share common signaling pathways, such as the inhibition of adenylyl cyclase, making the functional significance of expressing more than one SSTR subtype in the same cell unclear [2]. Whether the different SSTRs subserve different biological roles in the same cell or cooperate through dimerization to create greater signaling diversity remains to be determined. In this regard, we have recently shown that SSTR1 and SSTR5 heterodimerization, in stably transfected HEK and CHO-K1 cells, results in a new receptor with enhanced signaling properties [48,49]. We further anticipate such a possibility of heterodimerization between SSTR1 and SSTR5 and, additionally, between SSTRs and ErbBs in breast cancer cells.

Whereas SSTRs have been associated with antiproliferative signaling, several previous studies, using a variety of tumors including MCF-7 and MDA-MB-231 cells, have correlated ErbBs with tumor progression and poor prognosis [19,22,50,51]. However, the data have been inconsistent and controversial [52-54]. These inconsistencies may have arisen due to the techniques employed, the variation between cell stocks studied in different laboratories and, most significantly, the different passages at which the cells were used [45]. In this regard, we have seen significant variation in receptor expression/levels at different passages (data not shown). In keeping with ErbBs roles in tumor progression and poor prognosis, overexpression of ErbBs in breast carcinomas has been correlated with a lack of ER [44,52]. Furthermore, blocking ER using antisense strategies resulted in increased ErbB1, no change in ErbB2 and a slight decrease in ErbB3 expression in breast cancer cells [22]. Consistent with these observations, we found higher levels of expression of ErbB1 and decreased levels of ErbB3 in ERα – (MDA-MB-231) than in ER+ (MCF-7) cells. In accordance with previous studies, our findings strongly support the concept that the presence of ER could be a determining factor in ErbB expression in both breast cancer cells and tumors.

Previous reports state that specific ErbB heterodimers, i.e., ErbB1/ErbB2 and ErbB2/ErbB3, result in increased tumor growth and cell proliferation. We report that, in MCF-7 and MDA-MB-231 cells, there is preferential colocalization of ErbBs with other ErbBs. We found greater colocalization between ErbB1 and ErbB3 in both MCF-7 and MDA-MB-231 cells. We also detected a high degree of colocalization between ErbB2 and ErbB4 in MCF-7 cells. These data strongly support previous observations whereby heterodimerization between ErbB1 and ErbB2 was correlated with tumor progression [22,51]. These alternate heterodimer pairs, i.e., ErbB1/ErbB3 and ErbB2/ErbB4, may account for the less aggressive proliferation rates reported for both cell lines. Furthermore, in agreement with previous studies, we detected fewer cells showing ErbB colocalization in ERα – cells (MDA-MB-231) than in ER+ (MCF-7) cells with the exception of those coexpressing ErbB1 and ErbB3. Altogether, the higher degree of colocalization of ErbBs in MCF-7 cells than in MDA-MB-231 cells may be partially associated with slower tumor growth and better response to hormonal therapy. Our data provide direct evidence that ErbB1 and ErbB3 are the prominent subtypes which may interact as heterodimers, in these cells. Nothing is currently known regarding the physiological responses and functional consequences of these observations suggesting that further studies are required in this direction.

In addition to heterodimerization within receptor subfamilies, there have been several reports demonstrating that crosstalk between RTKs and GPCRs modulates downstream signaling pathways [35-37]. Even so, direct evidence for functional interactions between ErbBs and SSTRs have not yet been demonstrated despite the critical roles they play in tumor progression. We showed here that there was increased colocalization of SSTRs with ErbBs in MCF-7 cells (ER+) compared with MDA-MB-231 (ERα-) cells. This may help elucidate why estrogen-sensitive tumors show less aggressive proliferation than estrogen-insensitive tumors. This pattern of colocalization may also explain the superior response of ER+ patients to SST analog therapy [55]. In MCF-7 cells, the preferentially greater colocalization of SSTRs with ErbB2 may serve to counteract any deleterious effects of ErbB2. Whether this colocalization exists *in vivo *and is lost during tumor progression needs to be determined. Furthermore, colocalization of SSTR1 and SSTR5 with ErbB4 supports the antiproliferative effects of both SSTRs. SSTR interactions with ErbB4 may also serve to potentiate ErbB4's previously reported role in differentiation and apoptosis [30]. Furthermore, by preventing ErbB4's downregulation, SSTRs may be indirectly circumventing ErbB1-3's growth promoting effects. However, whether such interactions exist *in vivo *in solid tumors needs to be determined.

Despite SSTR and ErbB colocalization, low abundance of SSTRs alongside high expression of ErbBs within the same cell may account for the failure of SST treatment of breast tumor or other ErbB-expressing tumors. Furthermore, it is anticipated but not yet proven that SSTRs would reverse the effects of ErbBs with respect to MAPK activation and subsequent cell proliferation [56-58]. In addition, some reports suggest that the ER is involved in MAPK activation [59-61]. Previous studies have also demonstrated that ER presence is required for cbl-induced ubiquitination of ErbB1 and that ubiquitination of ErbB1 results in its degradation [62]. This could result in different levels of activation of downstream pathways in ER+ (MCF-7) and ERα – (MDA-MB-231) breast cancer cells. In addition, SST-induced internalization and subsequent downregulation of SSTR2-5 on the membrane may release ErbBs from complexes and result in cell proliferation [63-65]. Altogether, this suggests that not only do we need to activate SSTRs to counteract ErbBs effects on cell proliferation but we also need a mechanism to upregulate, or at least maintain, SSTRs on the membrane in order to reduce or modify ErbB signaling.

## Conclusion

In summary, the present results have important functional and therapeutic implications. Predominant SSTR1 expression and weak SSTR5 expression in breast cancer cells may help explain their poor sensitivity to hormonal therapy. These data may also explain the differential effects of the SST analog octreotide in breast cancer therapy. Since there is evidence of crosstalk between GPCRs and RTKs, cells displaying SSTR colocalization with ErbB suggest that, within these cells, both receptor families may functionally interact through hetero-oligomerization. If such a process exists, it may account for the diversification of receptor signaling. Most significantly, developing a new therapeutic agent that could both activate SSTRs and inhibit ErbB overexpression could potentially be a way to block tumor progression.

## Materials and methods

### Materials and reagents

RPMI 1640 and L-15 culture media were purchased from Invitrogen (Burlington, Ontario). Fetal bovine serum (FBS) and Antibiotic-Antimycotic solution were purchased from Wisent (St. Bruno, Quebec). The protease inhibitor cocktail used for protein extraction was supplied by Sigma-Aldrich Canada Ltd (Oakville, Ontario). Normal goat serum (NGS) was purchased from Vector Laboratories (Burlington, Ontario). Polyclonal rabbit anti-SSTR antibodies were developed in the lab and their specificity has been previously described [66,67]. Purified mouse anti-ErbB1 (sc-101), ErbB2 (sc-08), ErbB3 (sc-7390), rabbit anti-ErbB1 (sc-03), ErbB2 (sc-284), ErbB3 (sc-285), ErbB4 (sc-283) and goat anti-ErbB4 (sc-283-G) were purchased from Santa Cruz Biotechnology (Santa Cruz, California). The secondary FITC- and Cy3-conjugated goat anti-mouse or anti-rabbit and Cy3-conjugated donkey anti-sheep IgG antibodies were obtained from Jackson ImmunoResearch Laboratories (West Grove, Pensylvania).

### Cell culture

MCF-7 cells were maintained in RPMI 1640 medium supplemented with 0.35 μM insulin, 10% (v/v) FBS and 1% (v/v) Antibiotic-Antimycotic solution at 37°C in an atmosphere of 5% CO_2_/95% air. MDA-MB-231 cells were maintained in L-15 medium supplemented with 10% FBS and 1% Antibiotic-Antimycotic solution at 37°C in flasks with phenolic caps.

### Expression of SSTR1-5 mRNA in MCF-7 and MDA-MB-231 breast cancer cells

SSTR1-5 and ErbB1-4 mRNA levels were measured by semi-quantitative RT-PCR in MCF-7 (ER+) and MDA-MB-231 (ERα-) breast cancer cells as previously described with some modifications [15,68]. Briefly, 5 μg of DNA-free RNA was reverse transcribed and the resulting cDNA samples were amplified by PCR using the following primers:

hSSTR1 forward 5'-TGGTGGGCTTCGTGTTGT-3'

reverse 5'-GATGACCGACAGCTGACTCA-3'

hSSTR2 forward 5'-ATCTGGGGCTTGGTACACAG-3'

reverse 5'-GAAGACAGCCACCACGAT-3'

hSSTR3 forward 5'-TCATCTGCCTCTGCTACCTG-3'

reverse 5'-TTGAAGCGGTAGGAGAGGAA-3'

hSSTR4 forward 5'-CGCTCGGAGAAGAAAATCAC-3'

reverse 5'-CCCACCTTTGCTCTTGAGAG-3'

hSSTR5 forward 5'-CTCTCTCTGGACCTTGTGCC-3'

reverse 5'-ACGAGCAAACAGGTACGCTT-3'

hErbB1 forward 5'-AGTCGCCCAAAGTTCCGTGAGT-3'

reverse 5'-TGGGAGGAAGGTGTCGTCTATG-3'

hErbB2 forward 5'-AACTCACCTACCTGCCCACCAA-3'

reverse 5'-GTGGTATTGTTCAGCGGGTCTC-3'

hErbB3 forward 5'-CAGGTCTACGATGGGAAGTTTG-3'

reverse 5'-CTCACGATGTCCCTCCAGTCAA-3'

hErbB4 forward 5'-ACCCTTCAGCACCCAGACTACC-3'

reverse 5'-GACCACCAGAGAAAGAGAGGGG-3'

β-actin forward 5'-ATCATGAAGTGTGACGTGGAC-3'

reverse 5'-AACCGACTGCTGTCACCTTCA-3'

The PCR products were separated by electrophoresis on 1.5% agarose gels stained with ethidium bromide, visualized under UV illumination and photographed using an Alpha Innotech FluorChem 8800 (Alpha Innotech Co., San Leandro, CA).

### Western blot analysis

Crude membrane extracts from MCF-7 and MDA-MB-231 cells were prepared using a glass homogenizer in 20 mM Tris-HCl, pH 7.5 (1:300 protease inhibitor cocktail) as previously described [69]. Membrane protein (25 μg) was solubilized in Laemmli sample buffer containing 62.5 mM Tris-HCl (pH 6.8), 25% glycerol, 2% SDS, 0.01% bromophenol blue and 5% β-mercaptoethanol. Samples were placed in boiling water for 5 min and fractionated by electrophoresis on a 10% SDS-polyacrylamide gel as described by Laemmli [70]. The fractionated proteins were transferred by electrophoresis to a 0.2 μm nitrocellulose membrane (Trans-Blot Transfer Medium, Bio-Rad) in transfer buffer consisting of 0.025 M Tris, 0.19 M glycine and 15% methanol. Western Blot analysis was performed as previously described with slight modifications [71]. Briefly, membranes were blotted with anti-SSTRs polyclonal (dilution 1:400) and anti-ErbB polyclonal (dilution 1:600–1500) antibodies. Blocking of membranes, incubation with primary and secondary antibodies and detection by chemiluminescence were performed with the WesternBreeze^® ^kit according to manufacturer's instructions. Molecular weights were estimated using the MagicMark XP Western Protein Standard (Invitrogen). Images were captured using an Alpha Innotech FluorChem 8800 gel box imager.

### Immunocytochemistry

MCF-7 and MDA-MB-231 cells were plated on glass coverslips in 24-well plates and processed for indirect immunofluorescence for colocalization as previously described with slight modifications [16]. Cells were washed once in PBS and fixed with 4% paraformaldehyde on ice for 20 minutes. After two subsequent washes in PBS, cells were incubated with 5% NGS (diluted in PBS) for 1.5 hours followed by incubation with SSTR (1:500) and ErbB (1:150) antibodies in 1% NGS (in PBS) for 48 h at 4°C. Cells were then washed twice in PBS followed by incubation with Cy3-conjugated goat anti-mouse (1:500) or Cy3-conjugated donkey anti-sheep (1:500) and FITC-conjugated goat anti-rabbit (1:100) secondary antibodies for 3 hours. After two subsequent washes in PBS, cells were mounted and viewed under a Leica DMLB microscope attached to a CoolSnap CCD camera. Adobe Photoshop was used, in a consistent manner, to create the overlays and to adjust the contrast and brightness of all images.

### Quantitative analysis

Counting of SSTR-, ErbB- and SSTR+ErbB-positive cells was performed directly at high magnification (40×) under a Leica DMLB microscope. At least 8 horizontal and 8 vertical fields per coverslip were randomly selected for each receptor combination. Total number of cells positive for either one or both receptors was considered as 100% and percent colocalization was calculated accordingly. Total number of cells counted per coverlip ranged from 205 to 877.

## Abbreviations

SSTR, somatostatin receptor; ErbB, epidermal growth factor receptor; ER, estrogen receptor; SST, somatostatin; GPCR, G protein-coupled receptor; RTK, receptor tyrosine kinase; MAPK, mitogen activated protein kinase; FBS, fetal bovine serum; NGS, normal goat serum

## Authors' contributions

HLW carried out all experiments, participated in the design of the study, performed the statistical analysis and helped to draft the manuscript. UK conceived the study, participated in its design and helped to draft the manuscript.
